# High-Performance Integrated Virtual Environment (HIVE) Tools and Applications for Big Data Analysis

**DOI:** 10.3390/genes5040957

**Published:** 2014-09-30

**Authors:** Vahan Simonyan, Raja Mazumder

**Affiliations:** 1 Center for Biologics Evaluation and Research, Food and Drug Administration, Silver Spring, MD 20993, USA; 2 Department of Biochemistry and Molecular Medicine, George Washington University, Washington, DC 20037, USA

**Keywords:** big data, bioinformatics, high-performance cloud computing, high-throughput sequencing, next-generation sequencing, genomics

## Abstract

The High-performance Integrated Virtual Environment (HIVE) is a high-throughput cloud-based infrastructure developed for the storage and analysis of genomic and associated biological data. HIVE consists of a web-accessible interface for authorized users to deposit, retrieve, share, annotate, compute and visualize Next-generation Sequencing (NGS) data in a scalable and highly efficient fashion. The platform contains a distributed storage library and a distributed computational powerhouse linked seamlessly. Resources available through the interface include algorithms, tools and applications developed exclusively for the HIVE platform, as well as commonly used external tools adapted to operate within the parallel architecture of the system. HIVE is composed of a flexible infrastructure, which allows for simple implementation of new algorithms and tools. Currently, available HIVE tools include sequence alignment and nucleotide variation profiling tools, metagenomic analyzers, phylogenetic tree-building tools using NGS data, clone discovery algorithms, and recombination analysis algorithms. In addition to tools, HIVE also provides knowledgebases that can be used in conjunction with the tools for NGS sequence and metadata analysis.

## 1. Introduction

The development of Next-generation Sequencing (NGS) technologies has provided the ability to produce enormous amounts of sequence data inexpensively, with up to a billion reads produced per run [1]. Massively parallel technologies and state-of-the-art platforms for genomic sequencing, namely Roche/454, Illumina/Solexa Genome Analyzer, and Applied Biosystems/SOLiD System have made great progress compared to Sanger’s traditional sequencing methods and, therefore, have rapidly transitioned to become the dominant genomic approach due to increased cost-effectiveness and higher throughput [2,3]. Anticipated third-generation sequencing technologies will further continue to increase the exponential flood of data and decrease the costs of sequencing [4]. Due to the increased high-throughput capacities of sequencing machines, the major challenge of NGS lies in bioinformatics and computational analysis of the data [4]. The computational bottlenecks have posed an urgent need for the genomics industry to develop quick and efficient methods to process this data in a highly parallelized fashion while retaining the integrity and traceability of data, annotations and computations.

High-throughput sequencing technologies has stimulated massive research in genomics resulting in petabytes of scattered NGS data, which is largely dispersed in archives and databases unavailable to the larger research community. The increase in the volume of raw sequence data from NGS technologies poses challenges to storage availability as genomic centers continue to increase sequencing throughput [5]. Outputs of a wide range of experiments, including sequencing reads, clinical trial data, and medical records, produce complex and heterogeneous biological data [6]. Better access to this information is vital for the scientific/regulatory/medical community to efficiently utilize this knowledge and achieve significant progress in health science, disease diagnosis, and personalized medicine. Bioinformatic analysis tools previously used for Sanger sequencing data are not able to handle complexities in data produced by new technologies due to performance limitations and differences in the type of data, such as shorter read lengths, high error rates, and vast amount of reads produced [5]. The bioinformatic community must keep pace with the increasing deluge of raw sequence data from NGS technologies. Therefore, the rapid data generation from sequencing machines should be coupled with development of efficient and flexible sequence analysis, processing and storage algorithms and platforms [7].

HIVE, the High-performance Integrated Virtual Environment, addresses issues associated with the increasing complexity of NGS data. HIVE is a cloud-based infrastructure which facilitates secure storage, annotation and computation of large-scale data through a web-driven interface. HIVE delivers scientists broad access to diverse data types in a standardized, accessible manner, and provides sophisticated tools for data visualization and computational analysis. This large-scale analysis infrastructure serves as a foundation for scientists to integrate modeling, simulation, experimentation, and bioinformatics. Unlike existing tools, HIVE can be considered as a bioinformatics operating system (OS) level product that delivers the full spectrum of application building capabilities to support the entire lifespan of NGS and related Big Data. Our emphasis compared to existing tools include seamless integration, hierarchical sharing, highly secure object traceability and auditing, novel native NGS algorithms, parallelization, biocuration, and United States Food and Drug Administration (FDA) regulatory compliance.

HIVE provides a secure, web-accessible portal for registered users to manage and compute on NGS associated data, as well as the means to visualize the outputs in a variety of graphical representations. This high-throughput computing infrastructure provides an enhanced execution cloud environment that virtualizes services. Due to a sophisticated sorting schema, the system can move computations to the data, a process which is less strenuous for hardware and network infrastructure than transferring individual data chunks to computation nodes. In addition to enhancing well-known industry standard NGS tools, HIVE also contains a novel, native toolbox for NGS analysis (such as the alignment tool, variant-calling tool, and several others). Further, the HIVE team has developed the novel honeycomb engine which implements a secure hierarchical control that regulates user access and privileges in a highly granular manner. This infrastructure will facilitate a means for scientists to conduct both efficient and secure NGS analysis.

Because the amount of NGS data is expected to grow exponentially as the industry develops, the design of hardware, software and network resources should account for scalable expansion. HIVE’s utilization of a Cloud Control Server provides a flexible and scalable system, and it allows of the system’s OS to maintain functionality when changed in size or volume. In the future, scalability of the HIVE system can be provided by implementation of the MetaHIVE concept where multiple HIVE instances (in different geographical locations) inter-communicate metadata information about the availability of resources, users and data. A single MetaHIVE portal will serve as an entry point while redirecting actual storage and computation requests to the nodes which are most readily available. This will not only reduce the load to any particular HIVE-node but will also relieve the need for extra-large data transfers and long compute queues.

There are currently two major HIVE infrastructures, which include public and private deployments of the platform. The George Washington University hosts the public instance [8], and the FDA hosts the private instance within their secure network, which can serve as a model for a private HIVE deployment in other organizations. This article summarizes the recent tools, algorithms, and projects developed by the HIVE team.

## 2. HIVE Key Features

### 2.1. Data Transfer and Storage

**Figure 1:**
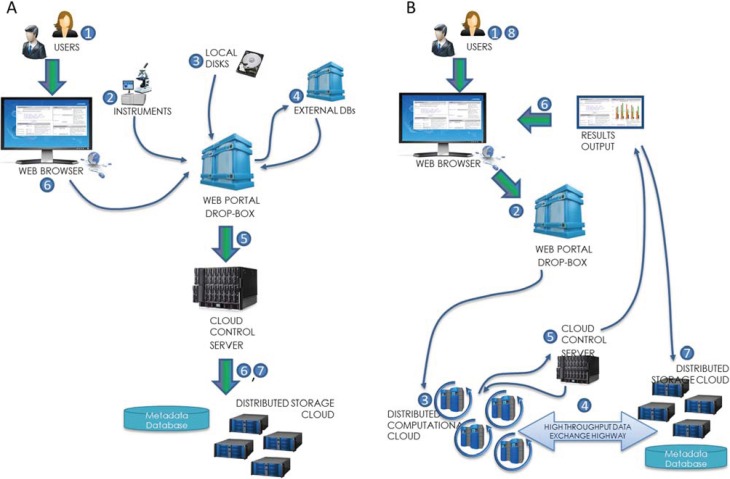
(**A**) Data Loading. Users may initiate data loading from local disks, external databases, or directly from scientific instruments. Downloads and uploads are split, monitored, concatenated and validated by the cloud control server during collection. Data is compressed, encrypted, indexed and archived in the distributed storage cloud and metadata database; (**B**) Computations. Users configure the analysis from the web browser interface by selecting the data inputs, choosing the desired algorithm and specifying parameter values. Processes are split and distributed among compute nodes after the data is retrieved from storage. Computations are monitored and the cloud control server coagulates parallel outputs. When results are complete, summaries and visualizations are sent back to the web browser.

The rate of genome sequencing has outpaced Moore’s Law, and it is anticipated that the output rate will continue to grow exponentially in coming years [9]. One run from a sequencing machine on a single sample can take >200 GB space, and much more when parsed and annotated. HIVE has the ability to load and store large-scale and heterogeneous datasets in the range of petabytes. Panel A in Figure 1 displays the data loading process in the HIVE system. Users may initiate data loading from local disks, via search and retrieval from external database URLs (such as NCBI, UniProt, *etc*.), or directly from scientific instruments. Downloads and uploads are split, monitored, concatenated and validated by the cloud control server during collection. Extant metadata is automatically harvested, however, additional metadata may also be manually provided by the user upon initiation of the download or after the data is uploaded. Formats are recognized and converted into internal standards for optimal efficiency within the system. Data is compressed, encrypted, indexed and archived in the distributed storage cloud and metadata database as appropriate.

**Figure 2:**
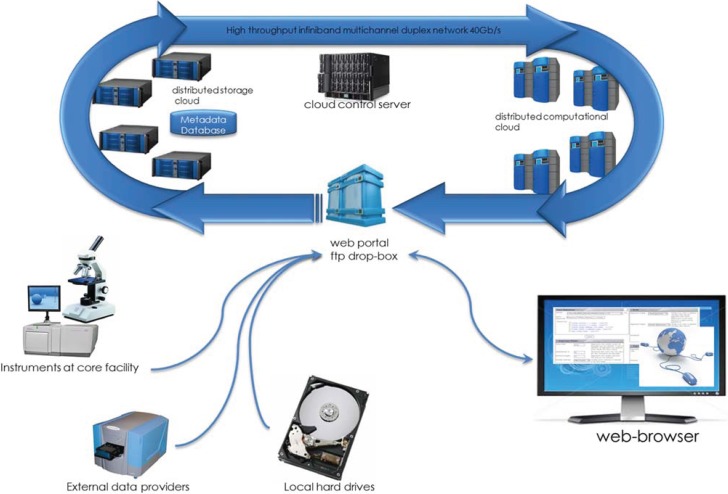
HIVE Topology. HIVE consists of a central cloud control server, seamlessly linked to both distributed storage and computational clouds. Data transfer bottleneck challenges are minimized with the location of the storage system and the database of sequence read archive metadata on the same network. The distributed storage layer of software and drivers is the key component for file and archive management and the backbone for the deposition pipeline. The data deposition backend adds the capability to automatically download and update external data sets to HIVE data repositories.

The high dimensionality of NGS and other high-throughput data has created many challenges with storage and integration of the data, and data transfer is one of the major bottlenecks associated with large volumes of sequence data [10]. In HIVE, the distributed storage system and database of sequence read archive metadata are located on the same network, which reduces input/output (I/O) and data transfer bottlenecks. Indexing and storage mechanisms facilitate high compression rates for archiving rarely used data, while providing easy access to actively used datasets. Figure 2 displays an overview of HIVE topology. The internal implementation design encompasses a metadata infrastructure that represents a hierarchy of interconnected database tables. This database schema is optimized for parallel queries allowing scalability as data amount and type increases. The model provides the benefit of high level parallelization found in relational databases for storage of and access to the data, while simultaneously exposing a simple, standard virtual distributed file-system on disk, for straightforward access to the data by algorithmic, computational, visualization, and data transformation data processing modules.

### 2.2. Computations

Due to the sheer volume of data and short read lengths of next-generation sequencing, there are many computational challenges associated with alignment, assembly, and analysis of the reads produced from sequencing experiments [11]. A few obstacles to bioinformatics analysis include repetitive regions in DNA sequences, sequencing errors from NGS technologies, and large volumes of short DNA fragments requiring assembly [11,12]. The HIVE platform provides a massively parallel computational backbone that integrates tools through generic adaptation mechanisms while providing interfaces and access to the results of the computations by standardized means.

Panel B in Figure 1 displays the computation process in the HIVE system. Users configure the analysis from the web browser interface by selecting the pre-loaded data inputs, choosing the desired algorithm and specifying parameter values. The request is submitted through the web portal and automatically parallelized based on the most optimal execution strategy depending on the input data. Parallel processes are subsequently executed in the distributed computational cloud after the correct data is retrieved from the distributed storage cloud. Computations are monitored and the cloud control server coagulates parallel outputs when and if needed. When results are complete, summaries and visualizations are sent back to the web browser. A copy of the results is also sent back to the distributed storage cloud for archival.

### 2.3. Security, Permissions and Auditability

Computational pipelines for NGS data must not only provide for the rapid bioinformatic analysis of large datasets, but must also offer secure storage and processing of genomic, proteomic, and metabolomics datasets [13]. Tracking data access by individual users in clinical large-scale research projects is often lacking in existing bioinformatic systems [14]. However, clinical research data, algorithms, processing pipelines, and generated results are sensitive and proprietary in nature, whether this is due to regulatory data, patient data, or pre-publication research. Access to such systems should be strictly controlled. All access to and manipulations of secured objects in HIVE are logged and available as auditable reports. Different objects and metadata in the system have a wide range of visibility depending on the hierarchical group membership of the users. Owners of files and computational results strictly control the access to these items in a very granular manner by defining data privileges for other users and member groups.

**Figure 3:**
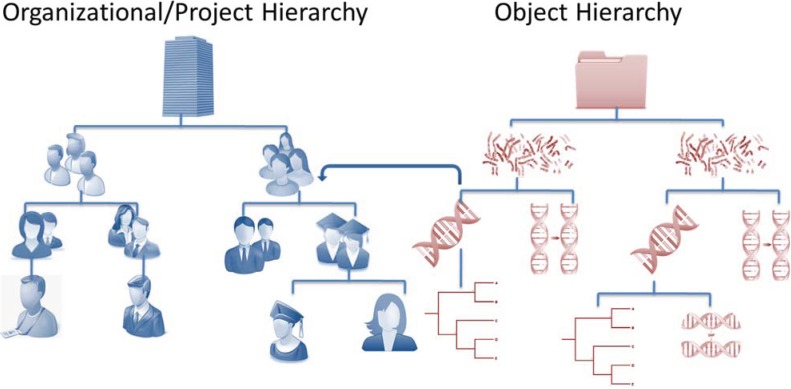
The Hierarchical Security Mapping System. The honeycomb engine implements a secure hierarchical control system that regulates user access and privileges in a highly granular manner. Hierarchically organized objects are assigned permissive or restrictive attributes relative to a particular user or branch, which can be inherited down to a group’s members hierarchically or propagated up to higher level management.

Due to the collaborative nature of scientific research, data accessibility and usability raise challenges concerning the owners of primary data and possible restrictions that should be applied to downstream use and analysis [15]. The novel honeycomb engine developed for HIVE provides an extremely secure hierarchical access control and permission system, which is designed specifically for sensitive data. Figure 3 displays HIVE’s hierarchical security mapping system, which assigns membership privileges to users, as well as establishes organizational and/or project hierarchies. The memberships assigned to users can audited and confirmed. Hierarchically organized objects are assigned permissive or restrictive attributes relative to a particular user or a branch. Access privileges may be inherited down an organization’s members, or propagated up to higher-level groups of administrators and supervisors.

### 2.4. Visualizations

Visualization in bioinformatics provides researchers with an overview of large and complex biological datasets, assists in determining patterns and relationships within the data, and facilitates data mining and conceptualization of analysis results [16]. Interactive graphics allow scientists to visualize genomic data at higher levels than that offered by basic sequence data and simple text results [17]. HIVE toolkits provide an agile development infrastructure to generate high performance parallel algorithms, data driven web-applications, and multifaceted visualizations. The HTML-5 graphical engine provides a foundation for the graphical visualization package. In addition, graphical interfaces for analysis and export functionalities in multiple formats can be used to transfer results to other user tools for visualization and additional analysis.

**Figure 4:**
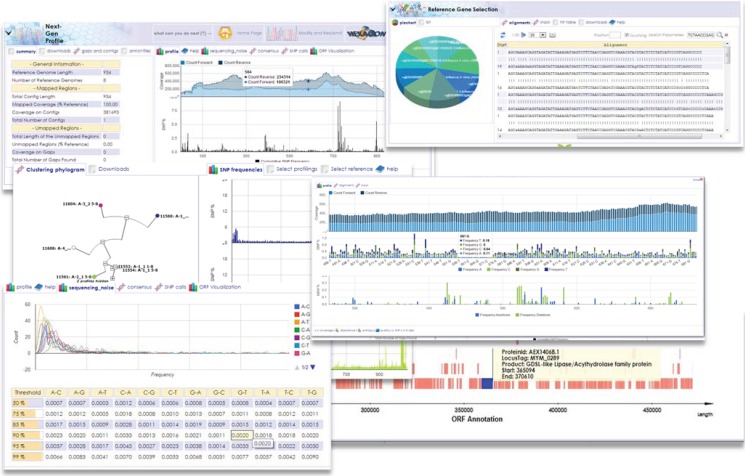
Multifaceted Visualizations in the HIVE Interface. Visualization tools for NGS analysis typically include viewers for genome and sequence annotations, sequence alignment results, molecular pathways, expression profiles, and hierarchical structures of ontologies, taxonomies, and phylogenies. The basic interface is designed to interactively select ranges of sequences, assign tags and categories and enter curated information describing those domains.

Generic sequence, annotation and visualization controls are a list of web-tools which allow the user to view sequence annotations obtained from remote resources or as a result of HIVE computational results. The basic interface is designed to interactively select ranges of sequences, assign tags and categories and enter curated information describing those domains. Visualizations tools for NGS analysis typically include genome and sequence annotations, sequence alignment results, molecular pathways, expression profiles, and hierarchical structures of ontologies, taxonomies, and phylogenies [16]. Figure 4 displays an array of visual outputs of computational analyses in HIVE.

## 3. Tool/App Development and Analysis

**Figure 5:**
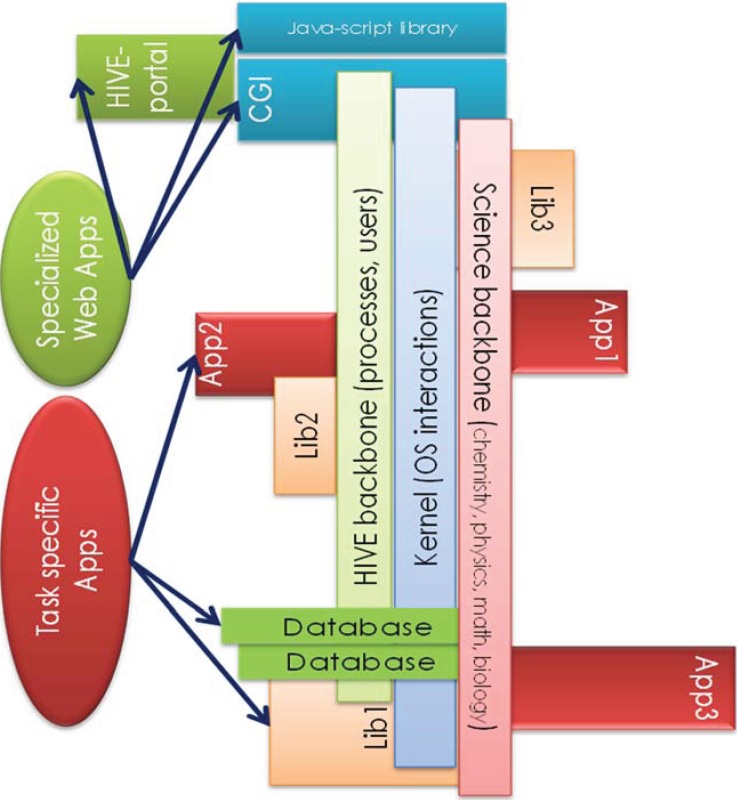
Integration of Tools and Datasets. The HIVE platform integrates and parallelizes widely used tools with ease. Tools integrated can be native applications developed by HIVE core team, but can also be developed by the community of researchers using Software Development Kits (SDK) accessing the system trunks and libraries through a set of versatile Application Programming Interface (API) functions.

HIVE provides an operating system to incorporate various software through a data analytics backbone. Tools integrated can be native applications developed by HIVE core team, but can also be developed by the community of researchers using Software Development Kits (SDK) accessing the system trunks and libraries through a set of versatile Application Programming Interface (API) functions. Figure 5 displays a schema for rapid integration of tools and datasets into HIVE, and subsequent development of novel applications on the HIVE backbone.

Currently, HIVE contains a combination of native tools developed specifically for the HIVE infrastructure and industry-standard tools that have been adapted and embedded within HIVE. The highly parallel processing backbone of the system allows enhanced compatibility and performance for both native and integrated industry-standard tools. HIVE has integrated various external bioinformatic tools which can operate on HIVE’s underlying infrastructure. Integration of external applications creates a fully customizable toolset that allows users to perform computations specific to their particular scientific research. HIVE contains data loaders, converters, and validators for industry-accepted data formats that include fasta, fastq, Sequence Alignment/Map (SAM), Binary Alignment/Map (BAM) files, *etc*.

### 3.1. HIVE Algorithms and Databases for Functional Analysis of Whole Genome Sequencing Data

**Figure 6:**
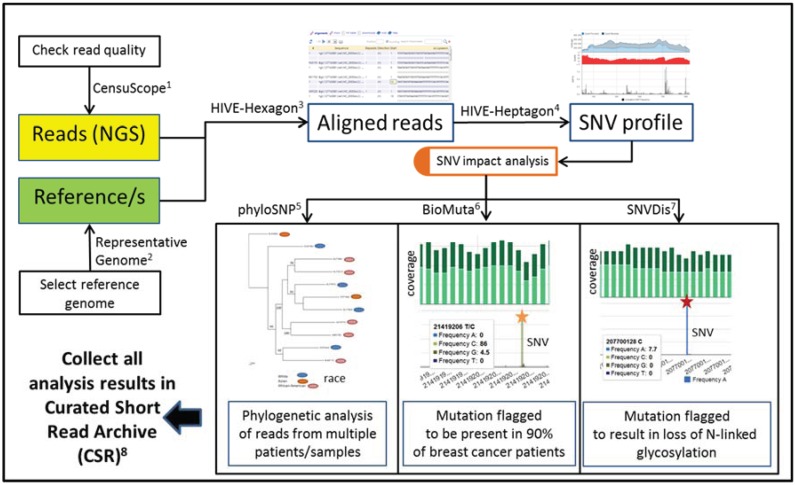
WGS Analysis Pipeline. Integration of multiple tools and databases allows for end-to-end analysis, which includes CensuScope read quality check, Representative Genome selection of a reference genome, HIVE-hexagon sequence alignment, and HIVE-heptagon SNP profiling. Further, users may choose to identify SNVs and analyze their functional impact through phyloSNP, BioMuta, and SNVDis.

HIVE is organized modularly to allow individual analyses to occur as discrete processes or to be stacked one after another in a workflow. An example functional analysis workflow of human data in HIVE is shown in Figure 6. This workflow displays the integration of multiple tools and database projects for stacked end-to-end analysis. The superscript numbers in Figure 6 correspond to specific tools as described in the text below. In this figure, we display a routine data analysis pipeline in HIVE, which includes CensuScope read quality check, Representative Genome selection of a reference genome, HIVE-hexagon sequence alignment, and HIVE-heptagon SNP profiling. Further, users may choose to identify SNVs and analyze their functional impact through phyloSNP, BioMuta, and SNVDis which has been described in previous publications from our team [18,19,20,21]. Additional HIVE tools for analysis of NGS data are also included in this section.

#### 3.1.1. CensuScope

CensuScope is a census-based metagenomic taxonomy analyzer for NGS data. This application provides a tool for quick assessment of biodiversity and taxonomic composition of metagenomic samples. Metagenomic datasets come from heterogeneous microbial communities containing thousands of species, and it is difficult to interpret the large, noisy, and partial data [22]. CensuScope provides an alternative solution to mapping millions of metagenomic reads to non-redundant databases, such as NCBI non-redundant nucleotide database (nt), which is computationally intensive and time-consuming. CensuScope estimates taxonomic composition of metagenomic samples through a subsampling-based algorithm. This natively-developed tool is complimentary to existing signature based tools, such as Pathoscope [23], Metaphyler [24], AMPHORA2 [25], and MetaPhlAn [26] because it allows the user to determine the content of a metagenomic sample with a fraction of the reads and iterations needed by similar tools and can use NCBI nt. This tool may be used as an initial Quality Control (QC) step to select optimal sets of reads before complete analysis. Reads from a metagenomic dataset are randomly picked using the Random Picker (RP) method, an algorithm developed specifically for CensuScope. The user decides on the number of reads and iterations to perform on the metagenomic dataset, which affects the length of time needed to run the analysis. The randomly selected reads are queried against NCBI nt using BLAST [27]. Taxonomic information from the hits is retrieved from NCBI taxonomy database. CensuScope can be easily modified to use a variety of index sequence, taxonomic classification, or mapping algorithm databases. Users may use CensuScope to observe how the short reads mapped to the NCBI taxonomy nodes, which provides an easy decision on the alignment strategy (the choice of reference, the need of filtration, *etc*.). Conversely, CensuScope can also be used after the short reads alignment (HIVE-hexagon) in order to focus on the detection of unaligned reads.

#### 3.1.2. Representative Genome

With the multiplicity of genomes available from individual species it can be difficult to identify the appropriate reference genome to use for mapping the reads. Representative Genome, a procedure developed by our group members in collaboration with PIR (pir.georgetown.edu) and other researchers, clusters closely related genomes based on their proteome similarity [28] which can be used in conjunction with CensuScope to discover which genomes the reads best match. Once identified, the specific genome can be used as a reference to map the reads.

#### 3.1.3. HIVE-Hexagon Alignment Tool

A major challenge in genomic workflows is alignment of short reads generated from high-throughput sequencing experiments to a reference genome. In fact, with the advent of NGS technologies it is no longer feasible to use BLAST to map millions of reads to the reference. Faster algorithms have been developed for this purpose and also for identifying variations with varying level of sensitivity and specificity [29]. The alignment step is computationally intense and complicated by the massive volume of short reads and variations in base quality [4]. Other difficulties during alignment include genetic variation in the population and sequencing error from NGS technologies [30]. HIVE-hexagon is a novel, massively parallel, ultra-fast mapping algorithm developed specifically for the HIVE platform [31]. HIVE-hexagon is the recommended alignment tool for the HIVE platform due to significant improvements in speed and sensitivity compared to other recognized alignment algorithms. Further, the HIVE-hexagon alignment tool utilizes the HIVE architecture and has been developed and optimized exclusively for use in the multi-component cloud infrastructure. Other well-known existing alignment tools have also been integrated into HIVE.

#### 3.1.4. HIVE-Heptagon SNP-Profiler

After alignment and mapping of short reads to a reference genome, the next step in genomic analysis workflows is variant calling. In this step, aligned sequences are compared to known sequences to discover positions that deviate from reference positions [4]. A major challenge in variation discovery is distinguishing true variation from sequencing errors and other machine artifacts [32]. The novel HIVE-heptagon SNP profiler plots the number of individual bases against the bases of the reference genome (or consensus sequence from prior outputs of the HIVE-hexagon) to identify SNPs and variation patterns. Each SNP that is called can be easily verified manually or programmatically for accuracy and traceability. Users can easily verify each variant discovered by the HIVE-heptagon tool since evidence for the call is stored in the HIVE system. Results from variant detection can be annotated to predict functional significance of changes, as well as compared with databases containing known associations with diseases [18,21,33].

#### 3.1.5. phyloSNP

Analysis of the impact of Single Nucleotide Variations (SNVs) is the next step in the workflow described in Figure 6. Comparative analysis of SNV profiles allows us to better classify patients, viruses or any other organisms from which the samples have been derived, and also provides clues to discovering genotype to phenotype connections. For example, in order to determine disease-causing SNVs, one first needs to classify the population and subsequently identify SNVs which are common in that population with similar outcomes. phyloSNP [34] is available as both an integrated tool in HIVE and a stand-alone application in which the user provides the SNV or variation dataset and the program produces a phylogenetic tree based on the SNVs present in the provided datasets. phyloSNP generates phylogenetic trees from SNV data from viruses to eukaryotes and can also take into account closely occurring SNVs without the need to use other programs for intermediate steps. In this way, phyloSNP proves superior to existing programs that construct phylogenetic trees from whole genome sequencing (WGS) data, such as AMY-tree [35] and SNPTree [36]. In addition, output files can be used as input files for other tools in the NGS analysis pipeline. The phyloSNP tool can be used for comparative genomic analysis of viruses, bacteria and eukaryotic species to aid in understanding distribution of variations across whole genome sequencing data. SNV Shrunk Genome is a function of phyloSNP that produces concatenated genomes around SNVs and provides sequence alignment as output. This tool allows the user to run an unknown sample against a reference genome and obtain an estimated genomic sequence for the sample, allowing for further phylogentic analysis using external tools.

#### 3.1.6. BioMuta

The Cancer Biomarker Variation Database, BioMuta [18], is part of an integrated Sequence Feature Database [18]. Functional information on each and every nucleotide base or amino acid (sequence feature) is critical for understanding the effects of variation. The organization of information is based on annotation of sequence features in UniProt which we have used for both the proteome and mapped to the genome with specific emphasis on the following columns: Organism name | Genome sequence file id | Key name | ‘From’ endpoint | ‘To’ endpoint | Source | Description. Disease names are curated using Disease Ontology to ensure standardization of information. The most widely used genome builds are chosen for mapping the features based on consultations with NCBI, EBI and UCSC and MODs. Sequence feature information in BioMuta is collected from various sources, including COSMIC [37], ClinVar [38], UniProtKB and through manual biocuration of publications. This database is updated continuously and available through public-HIVE via web-services and a simple tool, which takes in variation files in standard format and produces a mapping result which can be viewed in genome browsers in addition to integrating the table into HIVE for allowing end-to-end workflows. The database will support research, diagnostic development, and clinical biomarker discovery.

#### 3.1.7. SNVDis

Amino acid changes due to non-synonymous variation are included as annotations for individual proteins in UniProtKB/Swiss-Prot and RefSeq, which present biological data in a protein- or gene-centric fashion. Unfortunately, genome and proteome-wide analysis of non-synonymous single-nucleotide variations (nsSNVs) is not easy to perform because information on nsSNVs and functionally important sites are not well integrated both within and between databases and their search engines. The HIVE team has collected data from major variation databases (UniProtKB [39], dbSNP [40], COSMIC [37] and others) and comprehensive sequence feature annotation from UniProtKB, Pfam [41], RefSeq [42], Conserved domain database (CDD) [43] and pathway information from PANTHER [44] and mapped all of them in a uniform and comprehensive way to the human reference proteome provided by UniProtKB/Swiss-Prot. Integrated information of active sites, pathways, binding sites and domains, which are extracted from a number of different sources, give a detailed overview of how nsSNVs are distributed over the human proteome and pathways and how they intersect with functional sites of proteins. It is also possible to find out if there is an over- or under-representation of nsSNVs in specific domains, pathways or user-defined protein lists [19].

#### 3.1.8. Curated Short Read (CSR) Archive

Although biocuration is important for advances in biological discovery and biomedical research, data curation often lags behind data generated from sequencing machines in terms of funding, development and recognition [45]. Therefore, Big Data analysts often find a lack of curated information present in the primary NGS data repositories. The Curated Short Read Archive Project aims to curate metadata associated with publicly available short read sequences. The HIVE team has curated and analyzed prostate cancer data from the NCI cancer genomics program [46]. As proof-of-concept, we have analyzed single-nucleotide variation (SNV) and curated exome sequence data from 100 cancer controls and cases available from TCGA. We developed a workflow to analyze nsSNVs and curated metadata associated with TCGA samples. A current area of focus for this biocuration project is data from prostate cancer research. Sequences and metadata are obtained from the TCGA data portal [47], CGHub [48] and NCBI SRA [49]. The reviewed section has close to 200 sample results from more than 50 prostate and breast cancer cases and controls. SNV profiles can be compared based on phylogenetic analysis of the SNVs using phyloSNP to determine correlation between different cases and controls. Future goals for the CSR project include curating representative NGS data from cancer samples of all cancer types. HIVE provides the large-scale storage infrastructure for this project.

### 3.2. HIVE-Octagon Profile Clustering Tool

**Figure 7:**
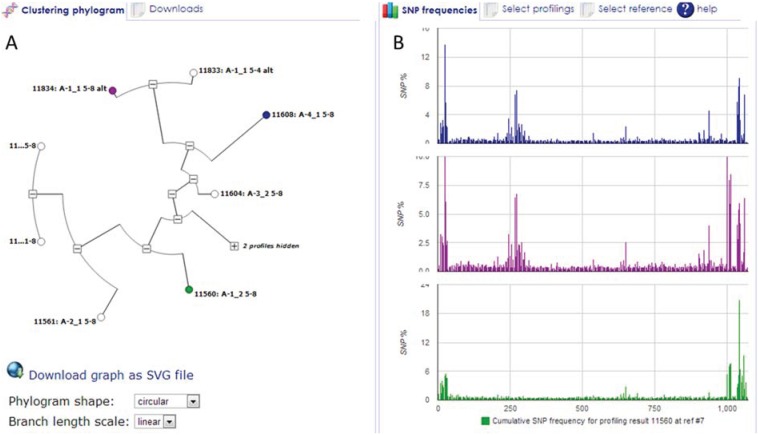
HIVE-octagon Profile Clustering Tool. This tool generates a sequence of SNP frequencies at reference genome positions, compares these sequences, and produces a hierarchical clustering which is displayed as an interactive phylogram. Panel A shows an interactive phylogram output, and Panel B displays graphs of SNP frequencies at reference positions for the three selected profiling results.

Output from the HIVE-heptagon SNV Profiling tool may be used as input for the HIVE-octagon profile clustering tool for comparative analysis of genomic sequence alignment profiling results. For each profiling result, this tool generates a sequence of SNV frequencies at reference genome positions, compares these sequences, and produces a hierarchical clustering which is displayed as an interactive phylogram. The tool offers several algorithms for comparing SNV frequencies and for calculating the clustering, as well as options for determining which frequencies and positions should be considered in the calculation, and which should be ignored. Profile pairs can be compared by a number of distance functions and subsequently fed through a clustering algorithm as specified by the user. All data and resultant outputs can be exported in a compatible format for additional analysis by external tools. This algorithm provides a detection method for identifying clusters of SNPs exhibiting similar patterns. Figure 7 displays a typical output of the HIVE-octagon Profile Clustering tool. Panel A shows an interactive phylogram output, and Panel B displays graphs of SNP frequencies at reference positions for the three selected profiling results.

### 3.3. Population Analysis Tool

The Population Analysis tool identifies patterns of mismatches between viral reference genomes and multiple reads coming from different isolates. RNA viruses replicate with extremely high mutation rates and display significant genetic diversity [50]. The dynamic mutation distributions that embody RNA viral populations are termed quasispecies [51]. The Population Analysis tool serves as a clone discovery tool, and analyzes next-generation data and plots predicted quasispecies evolution by linking mutations and building consensus sequences. The mutations are correlated as the tool scans through the reads by reading frame. The clone discovery algorithm correlates distant mutations in existing alignment data and identifies clones of low coverage. A visual interface of the output displays a Sankey diagram, which exhibits the coverage, position, and references. This diagram provides visualizations of the clones discovered. The Population Analysis tool analyzes a metagenomic sample of quasispecies, and it predicts the different species potentially present in that particular sample. The population analysis tool can aid in understanding evolutionary changes between genomes, designing diagnostics, and discovering treatment methodologies.

**Figure 8:**
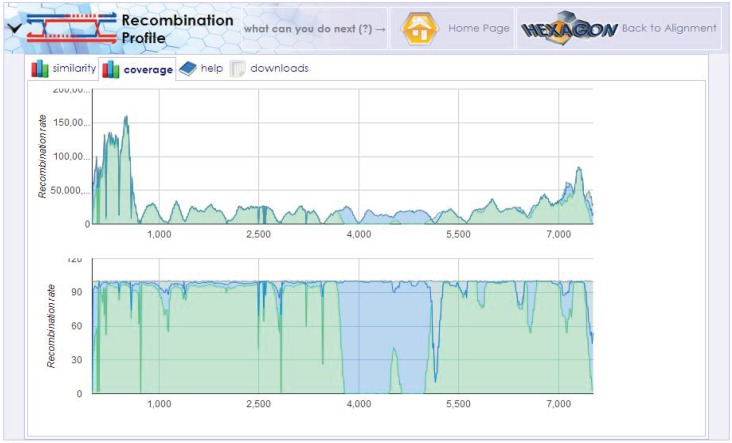
Reference Recombination Tool. This tool investigates the distribution of sample coverage across multiple reference genomes by comparison with a multiple alignment of the genomes. The example output displays three distinct reference genomes in the same biological sample, implying a recombination event.

### 3.4. Reference Recombination Tool

The Reference Recombination tool is designed to discover recombination events among related viral bacterial populations. Many microbes are known to recombine with different strains of the same species or participate in horizontal gene transfer from entirely distinct species to form completely new genomes. This tool uses previously computed mapping results of reads against mutual alignment of multiple reference genomes to determine the potential occurrence of recombination and outline significant recombination sites. Using components of a scientific visualization library, HIVE renders poly-plots of coverage distribution along the profiles of cross-genomic mappings. Figure 8 displays the Reference Recombination tool output, which shows the coverage of segments of three distinct reference genomes in the same biological sample, implying a recombination event.

## 4. External Tools

Currently, HIVE contains a combination of native tools developed specifically for the HIVE infrastructure described in the above sections, and industry-standard tools that have been adapted to and embedded within HIVE. The highly parallel processing backbone of the system allows enhanced performance for both native and integrated industry-standard tools.

### 4.1. External Aligners

HIVE has adapted various external alignment tools, such as BWA [52], Bowtie [53], BLAST and BLAT [54], and multiple alignment tools, such as CLUSTAL [55] and MAFFT [56]. Currently integrated *de novo* assembly tools include Velvet [57] and ABySS [58], and RNA seq tools include TopHat [59]. There are also capabilities to integrate additional external applications upon user request, which creates a fully customizable toolset that allows users to perform computations specific to their particular scientific research.

### 4.2. MetaGeneMark

MetaGeneMark [60] is an external algorithm integrated into HIVE for annotation of metagenomes. Sequence alignment outputs or non-annotated sequences in HIVE can be processed using the MetaGeneMark pipeline to identity open reading frames (ORFs). Assembled contigs can be used as input data in MetaGeneMark. The MetaGeneMark algorithm uses Hidden Markov models to predict genes from the supplied input sequence(s) [61]. All potential coding regions identified by MetaGeneMark are reported in a summary table on the HIVE interface. HIVE extends the utility of MetaGeneMark and directly links it to downstream analysis by querying coding region candidates against the NCBI sequence database using BLASTP to discover potential homologs. Hits from BLAST are summarized in a table viewer on the HIVE interface and organized by the original contig ID line. Hit information from BLAST consists of information for each corresponding region of interest, including position, length, GI and name of the candidate protein hit. Users can then verify or reject the results, save the annotations to the file, and subsequently commit the annotated sequence to the HIVE annotated representative database. Future plans include integrating UniRule for functional annotation [62,63].

## 5. Hive Operating Models

The scale of the hardware at which HIVE must be able to work varies from multi-thousand core supercomputer centers to HIVE-in-a-box at field offices with small compute-stations. HIVE can be installed in a variety of environments, including the following Operating Models: HIVE Enterprise, HIVE Service and HIVE-in-a-box.

### 5.1. HIVE Enterprise

The HIVE Enterprise approach provides a highly secure private cloud-operating model with internalized and restricted access within an organization or entity. The HIVE Enterprise solution can integrate seamlessly existing HIVE appliances, while providing its own computational storage and power. The implementation of a secure private cloud is critical for institutes such as hospitals, biopharmaceutical companies, or individual corporate entities that routinely handle personally identifiable information PII, sensitive patient information, and proprietary data. The HIVE enterprise system can be installed in High-performance Computing (HPC) centers driven by farm engines with hundreds of cores. Current working prototypes of enterprise installations of HPC environments include GW Colonial One (GW-HIVE) and FDA HPC (FDA-HIVE). The Enterprise system can have a configured number of computational nodes and storage nodes connected as a private network by a high-performance switch. Additional resources and the hardware base can be extended in a virtually unlimited manner.

### 5.2. HIVE-in-a-Box

It is conceivable that not all projects will require a full-fledged implementation of HIVE with extremely high computing and storage capabilities. HIVE-in-a-box serves as a pre-configured, portable appliance provided by HIVE developers. The optimization of software on a preconfigured and pre-deployed platform is critical to achieve maximum performance. This operating model is ideal for hospital, field office or laboratory usage, and provides an affordable alternative to the HIVE enterprise system. Software on the HIVE-in-a-box system is identical to the software present on the master HIVE server node, which includes the web front-end, database, and computational programs.

### 5.3. HIVE Service

HIVE Service provides an operating model in which users can easily access, compute, and scale-up analysis workflows on public cloud instances such those offered by Amazon. This operating model allows users to have immediate scalability for projects by providing a framework for renting both storage and computational capacity from public cloud service providers. Researchers with smaller projects may choose to rent capacity from cloud services instead of investing in informatics infrastructures (such as HIVE enterprise or HIVE-in-a-box). The HIVE Service operating model serves as an economic model for scientists or biologists in small research laboratories that need to perform analyses on a small number of sequencing experiments.

## 6. Industry Analysis

Several companies and groups are involved in providing Cloud Services, Workflow Management, and NGS Big Data infrastructures, each with their own emphasis.

### 6.1. Cloud Services

As mentioned above, not all projects require an Enterprise-level private cloud computing system. Projects which are smaller in scope or those which are being used as proof-of-principle for an analytic method should be able to leverage sufficient power from VMs. VMs can be rented from various providers allowing quick and easy deployment of pre-configured workflows in publicly available clouds. Cloud service providers, such as Amazon Web Services [64], Rackspace [65], Flexiant [66], and Google Cloud [67] offer users, researchers, scientists, and companies the means to rent hardware and storage and only pay the amount of allocated time, storage, or computational capacity [68]. These cloud services allow on-demand access to computational hardware and avoid upfront investments in computing hardware, maintenance and personnel [69]. In addition, downstream maintenance and scalability of hardware resources is no longer an issues for platform providers and users [70]. Ultimately, cloud services offer a new bioinformatic framework in which users can configure their own machine and storage infrastructure [70]. For example, Cloud BioLinux provides a platform for scientists and researchers to develop bioinformatics infrastructures by providing an assortment of pre-configured sequence analysis tools packaged within a high-performance VM server that runs on Amazon EC2 [71].

### 6.2. Workflow Management

Due to the increasing outputs of next generation sequencing data, workflow management systems are vital for efficient data integration and automating data analysis processes [72]. A considerable amount of development has been dedicated to developing workflow management graphical user interfaces (GUIs), such as Taverna [73], Galaxy [74] and others [75,76,77]. The Taverna Workflow suite allows for combining tools into bioinformatic analysis pipelines [73], and the Galaxy system allows users to create workflows from scratch in a graphical interface [74]. Other workflow management systems that aid in automating data analysis processes include Conveyer [78], Pegasus [79], Wildfire [80] and Kepler [81].

### 6.3. Big Data Infrastructures

Many groups like Galaxy, CLC Bio, Seven Bridges Genomics, DNAnexus, and UPPNEX are developing Big Data infrastructures with GUIs and integrated third-party applications for manipulation and analysis of genomics data. Here we briefly review these Big Data infrastructures and compare them to the HIVE platform. Feature emphasis for other platforms and software are based on information present in publications, websites and our usage experience.

Galaxy is a genomics data analysis platform that is available as a web-server and downloadable open source software for users [82]. Galaxy contains an interactive, graphical workflow editor that allows users to create workflows with ease and repeat analyses. Similar to HIVE, Galaxy is offered on the Amazon EC2 cloud with a “pay-as-you-go” operating model [83]. The two platforms differ with respect to user interfaces, including graphical visualizations, analysis outputs, and availability of tools.

CLC Bio is a bioinformatics software that offers command line tools and a GUI to analyze high-throughput sequencing data [84]. Similar to the HIVE platform, CLC Bio offers desktop software and enterprise solutions as operating models, however, CLC Bio does not allow users to define metadata. Because users can define metadata in HIVE, it is possible to create and integrate databases in HIVE within minutes which can immediately be used in workflows.

Seven Bridges Platform provides an environment for managing and executing NGS workflows [85]. Analogous to HIVE, Seven Bridges emphasizes data and process security. Further, Seven bridges is developing a “cloud-enabled appliance” which is similar to the HIVE-in-a-box operating model, [86]. Differences between HIVE and Seven Bridges include HIVE’s focus on development of novel algorithms for improved end-to-end NGS analysis.

DNAnexus allows for management and analysis of genomic data through a cloud platform [87]. Similar characteristics to HIVE include defined authentication and access controls, data and process security, and parallelization of computations. A major difference between the two platforms is that DNAnexus does not offer novel native NGS algorithms, tools and applications.

UPPNEX is an NGS infrastructure developed as a bioinformatic storage system and HPC environment [88]. Similar to HIVE, UPPNEX provides software and user support, computational pipelines and tools for NGS analysis. However, the platforms differ with respect to infrastructure. While the HIVE environment provides an easily accessible web interface for storage and analysis of NGS data, UPPNEX requires installation and maintenance of software for users. Further, HIVE provides a GUI on the web browser, while UPPNEX provides command-line interface for most of the installed software.

Overall, the HIVE platform delivers the full spectrum of capabilities for NGS and related Big Data lifespan. HIVE not only implements the wider set of tools but as an integration backbone can also be used to improve the functionality of other, above-mentioned software products by better amalgamation of data and software and by facilitation of distributed computations to advance the performance, maintainability, auditability and robustness of the existing solutions as a whole. In addition, the HIVE platform has more focus on data standardization and regulatory compliance efforts.

## 7. Lessons Learned

Although the HIVE web-portal and visualization engine of specific deployment instances discussed herein are newly developed within the past two years, the conceptual paradigms and developments empowering HIVE architecture began almost a decade ago. During that period of time, there have been many trials and errors, and many opportunities for learning experiences during the implementation of various solutions, some of which have been successful and others for which solutions are still being searched.

### 7.1. Disk Speed Limit and Network Connectivity Are the Primary Limitations

The quality of disk(s) and network connection entirely determine the performance ceiling. The quality or quantity of fundamental system components such as CPU, memory, *etc*. do not matter if the performance bottleneck is in data throughput: the system will only ever perform as fast as the disk and/or network allows. Thus, one should target investing in higher quality and higher density enterprise level storage units and multi-head network gear from the onset. Consider the situation where a 200 core system is operating with balanced distribution such that computational load is almost 100% across cores at any given time (meaning that CPUs are not waiting on data). In an attempt to improve performance, an additional 100 CPU cores could be added to the system. Contrary to one’s expectations, the system speed could significantly slow down due to network saturation. Jobs can now be split across more cores but the network must physically work harder to communicate with the additional cores and manage additional network communication overheads. Hence, there is an optimal balance of the network to cores ratio such that if either is saturated, system performance will be degraded.

### 7.2. All Code Should Be Considered Big Data Code

No matter how small the challenge for which a new code is being developed, developers should always assume Big Data applicability in a massively parallel platform and extension of usage to other contexts. Every wasted memory bit and CPU cycle repeated billions to trillions of times, when accumulated, can drastically inhibit efficiency in a high performance execution platform. Not only can particular data blobs be large, but the number of blobs or metadata records associated with a single task can be massive. This may cause conflict depending on operating system limitations restricting number of files/i-nodes, processes, *etc*. Smart and thoughtful planning of all newly developed code helps to alleviate waste of resources.

### 7.3. Bare Hardware Is the Better Choice Compared to Virtual in Terms of Efficiency/Speed

There are certain cases for which virtualization is well-suited, however, the size and complexity of typical NGS-related processes render virtual computers comparatively inefficient in terms of efficiency. Virtualization comes with an overhead cost, so even as it grants the ability to compute more processes concurrently on a single machine it comes with the tradeoff of losing compute power to the extent that execution can be halted completely. Virtualizer hosts interfere with optimization paradigms such as swap space, memory pagination and memory mapping used in efficient, modern operating systems. Absence of access to physical hard-drives and system hardware in a virtual execution environment results in layers of context switches between systems and damages the performance.

### 7.4. Knowledgeable Data Sorting and Localized Computation Execution Minimize I/O Problems

HIVE automatically recognizes and processes a number of file types according to properties and usage patterns of that type. Preprocessing, such as sorting, indexing and non-redundification, allows efficient transfer of packets of related information to a compute node where the individual information blobs are intended to be analyzed consecutively. Conversely, the model of virtualized services allows the transfer of the comparably small executable code to the location of the data instead of forcing the transfer of large chunks of information. This approach mitigates I/O inefficiencies by both decreasing the amount of internal data transfer that occurs and by optimizing cache loading.

### 7.5. There is No Efficient Method for Backup

Transfer and storage of Big Data already has enormous associated costs. By the time raw inputs are processed and indexed and results are computed, the amount of information relevant to a single computation can increase 10-fold. For now, HIVE has chosen to maintain only full backup of configuration data and metadata. Of course the needs of different users and organizations may dictate more comprehensive solutions, but the cost of maintaining duplicate copies of such large information remains a serious consideration.

## 8. Further Information

The HIVE implementation internal to the FDA network is already in use by teams of HIVE developers, scientists and regulatory personnel to optimize current methods and develop new approaches to NGS analysis. Furthermore, HIVE team members are working with FDA and key industry players to determine submission standards for NGS data and metadata. HIVE is rapidly becoming the platform of choice for Big Data within the FDA. Public-HIVE from Dr. Mazumder’s Lab at GWU conducts projects with researchers from several institutes. The public tools, applications and projects described are available at the GWU public HIVE website [8].
